# Pathophysiology and treatment strategies for COVID-19

**DOI:** 10.1186/s12967-020-02520-8

**Published:** 2020-09-15

**Authors:** Manoj Kumar, Souhaila Al Khodor

**Affiliations:** Research Department, Sidra Medicine, Doha, Qatar

**Keywords:** SARS-CoV-2, Pandemic, 2019 novel coronavirus, Viral inhibitor, ACE-2 receptor, Receptor binding protein

## Abstract

The outbreak of Coronavirus disease of 2019 (COVID-19) caused by the Severe Acute Respiratory Syndrome coronavirus 2 (SARS-CoV-2), has posed a serious health threat. The increasing number of COVID-19 cases around the world is overwhelming hospitals and pushing the global death toll to over 746,000, which has pushed the sprint to find new treatment options. In this article, we reviewed the SARS-CoV-2 pathophysiology, transmission, and potential treatment strategies.

## COVID19 pandemic background

Coronavirus Disease 2019 (COVID-19) caused by an infection with the Severe Acute Respiratory Syndrome Coronavirus 2 (SARS-CoV-2) has caused one of the largest global outbreaks in recent years, and posed a serious threat to the global public health [1, 2]. Considering the rapidly increasing cases of COVID-19 and disease severity, the World Health Organization (WHO) declared a global health emergency on January 30, 2020 [3]. Despite implementing worldwide combined efforts to prevent SARS-CoV-2 further transmission by quarantining the infected persons and their family members, social distancing, and schools closure, the spreading of infection could not be contained; therefore, on March 11, 2020, the WHO declared COVID-19 a pandemic [3]. As of now, around 213 countries and territories outside of the Mainland China have reported SARS-CoV-2 infections [1, 4]. The massive impact of SARS-CoV-2 infection has been seen in the United States of America, Europe, and Asia. As of Aug 12th, 2020, the time of writing this review, SARS-CoV-2 has infected more than 20.54 million people worldwide and resulted in 746,151 deaths (Additional file 1: Figure S1A).

The worldwide date indicates an exponential infection rate of SARS-CoV-2 cases after the first week of March-2020 (Additional file 1: Figure S1B). The mean primary reproduction number (R0) was estimated to range from 2.24 [95% confidence interval (CI) 1.96–2.55] to 3.58 (95% CI 2.89–4.39), and associated with two- to eight-fold increase in the reporting rate as compared to other viral infections (Additional file 1: Figure S1C) [5, 6]. The current statistics are showing that the epidemic doubling time is as low as 6.4 days [5], including potential asymptomatic transmissions. Although the situation is evolving and updated on daily basis, more data is required to confirm these estimations. This data indicates a high potential for the SARS-CoV-2 outbreak and warrants immediate therapeutic interventions.

### Outbreaks of coronavirus

**Fig. 1:**
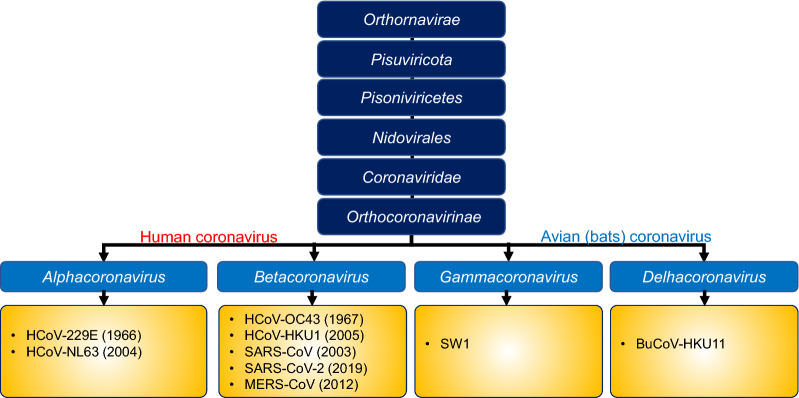
Taxonomy of Coronaviridae. HCoV, human coronavirus; MERS-CoV, Middle East respiratory syndrome coronavirus; SARS-CoV, severe acute respiratory syndrome coronavirus; SARS-CoV-2, severe acute respiratory syndrome coronavirus-2; SW1, Beluga whale coronavirus; and BuCoV-HKU11, bulbul coronavirus HKU11

Seven Coronaviruses (CoV) of zoonotic origins have crossed the species barrier so far, to cause infections in humans, and three of them have caused a deadly infection in last two decades, including the Middle East Respiratory Syndrome Coronavirus (MERS-CoV), Severe Acute Respiratory Syndrome Coronavirus (SARS-CoV), and SARS-CoV-2 (Fig. 1) [7–9]. Among these, SARS-CoV originating from bats emerged in Guangdong, China in 2002, and resulted in the 2003 outbreak with about 10% case fatality rate (CFR) [10], while MERS-CoV originating from the dromedary camels was first reported in Saudi Arabia in June 2012, and later in 27 countries, infecting a total of 2494 individuals and resulting in a CFR of about 34.4% [11]. The recent SARS-CoV-2 pandemic is thought to be originated from an animal reservoir, through spillover infection, before being transferred to humans in Wuhan city of China [12]. Although the exact mechanisms of SARS-CoV-2 transmission are not fully understood, human-to-human transmission of SARS-CoV-2 from patients or asymptomatic carriers occurs via two routes [13]. The first is directly through close contact with an infected person (< 2 meters) where respiratory secretions can enter, mouth, eye, nose, or airways. The second route is indirect, via the touching of an object, surface, or hand of an infected person contaminated with respiratory secretions and subsequently touching own’s mouth, eye, or nose [13]. The SARS-CoV-2 infection resulted in highly variable CFR depending on co-morbidity and country—ranging from 0.1 to 9.26% [14].

### SARS-CoV-2 Structure and Pathophysiology

**Fig. 2:**
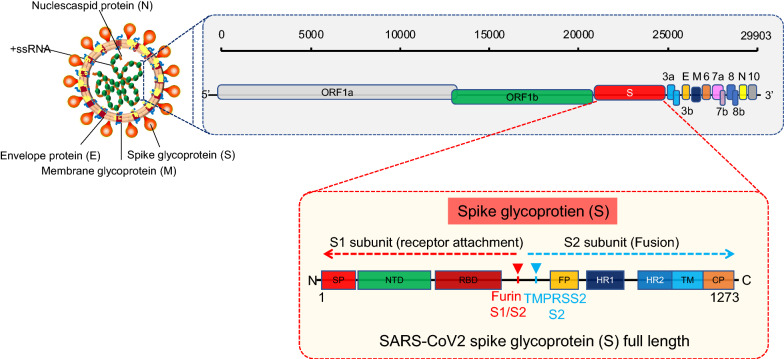
Schematic representation of the SARS-CoV-2 structure, genome and functional domain of SARS-CoV-2 S protein. The viral surface proteins, envelope membrane and spike, are embedded in a lipid bilayer, while the single-stranded positive-sense viral RNA (SS-RNA) is associated with the nucleocapsid protein. The spike proteins contain S1 and S2 subunits and the cleavage sites for furin and TMPRSS2. The spike proteins mediate the viral attachment to the host cells after activation by the enzyme TMPRSS2. SP, signal peptide; NTD, N-terminal domain; RBD, receptor-binding domain, contains core binding motif in the external subdomain; FP, fusion peptide; HR, heptad repeat 1 and heptad repeat 2; TM, transmembrane domain; CP, cytoplasm domain

To understand the pathogenic mechanisms of SARS-CoV-2 and to discuss the current therapeutic targets; it is important to describe the viral structure, genome, and replication cycle. CoVs are positive-stranded RNA viruses with a nucleocapsid and envelope [15]. A SARS-CoV-2 virion is approximately 50–200 nm in diameter [16] and has a +ssRNA genome of approximately 29.9 kb in length—the largest known RNA virus with a 5′-cap structure and 3′-poly-A-tail and possess 14 putative open reading frames (ORFs) encoding 27 proteins [17, 18]. The virion has four structural proteins, known as the S (spike), E (envelope), M (membrane), and N (nucleocapsid) proteins; the N protein holds the RNA genome, and the S, E, and M proteins together create the viral envelope [19]. The spike glycoprotein-S facilitates the virus attachment to the angiotensin-converting enzyme 2 (ACE2) receptor and fuses with the membrane of the host cell [19]. SARS-CoV-2 then uses serine proteases TMPRSS2 (transmembrane protease serine 2) for S protein priming, infecting the target cells [20] (Fig. 2). The spike proteins of SARS-CoV-2 contains two subunits; S1 receptor binding subunit and S2 fusion subunit, to mediate the virion binding to receptor protein and initiate membrane fusion. The S1 and S2 subunits are divided by the S cleavage site (Fig. 2). To facilitate virion attachment to receptor and fuses with cells membrane, the spike protein needs to be cleaved by cellular proteases from the S1/S2 cleavage site (Fig. 2). Interestingly, the molecular analysis of S proteins identified an insertion at S1/S2 site, which is absent in other SARS-CoV [21], though the importance of this insertion is still unknown, it seems that this unique insertion is providing a gain-of-function advantage for an easy cell infection and efficient spreading throughout the human host.

The viral RNA hijacks the host cell’s machinery to initiate the viral genome replication and polypeptides chain synthesis and form the replication-transcription complex (RCT) needed to synthesize the sub-genomic RNAs as well as structural proteins (envelope and nucleocapsid) (Fig. 2). The viral envelope has a crucial role in the viral assembly, release, and promoting viral pathogenesis [22]. However, the exact role of the many small viral peptides (e.g., those of glycoprotein subunits) has not yet been described. More research is needed to understand the structural characteristics of SARS-CoV-2 that underlie various pathogenic mechanisms.

### Susceptibility to SARS-CoV-2 infection

**Fig. 3:**
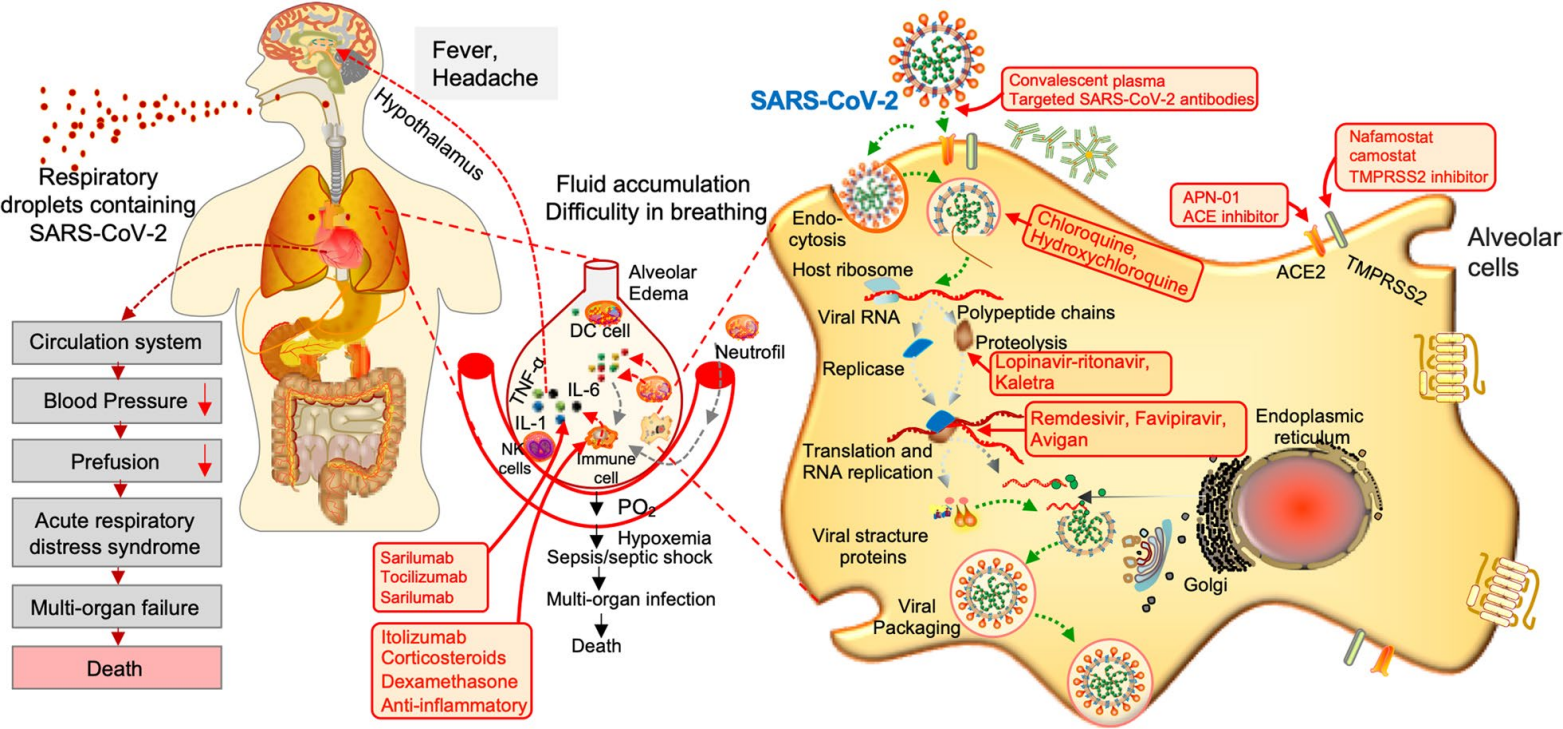
An infection and replication model of SARS-CoV-2 in host cells and current treatment strategies to interfere with steps in the SARS-CoV-2 replication cycle. SARS-CoV-2 binds to host cells through the ACE2 receptor, and after endocytosis and subsequent uncoating, the components of SARS-CoV-2 use host cells machinery to produce new viruses. Finally, the SARS-CoV-2 virions are released from the host cell by exocytosis. During this process, the viral replication can be inhibited at different stages by repositioned drugs (highlighted in red). On the other hand, SARS-CoV-2 stimulates the host immune system to release the cytokines and subsequent inflammation and immune-dysfunction through activation or impairment of various immune cells, such, dendritic cells, NK cells, macrophages, and neutrophils. This process can lead to sepsis, septic shock, multiple organ failure, and death. SARS-CoV-2, severe acute respiratory syndrome coronavirus-2; TMPRSS2, transmembrane protease serine 2; NK, natural killer; DC, dendritic cell; IL-1, interleukin-1; IL-6, interleukin-6

Symptoms of SARS-CoV-2 resemble those of the common cold, including fever, coughing, and shortness of breath [23]. However, the infection can lead to pneumonia, multi-organs failure, severe acute respiratory syndrome, and even death in severe cases (Fig. 3) [24]. Elderly individuals (aged > 60 years) and people with underlying chronic health conditions are more susceptible to severe disease (18.5%) as compared to children and younger healthy adults (6%) [25]. The clinical data collected from the non-survivors patients revealed that the most distinctive comorbidities of SARS-CoV-2 infection were hypertension (24–75%) and diabetes mellitus (16.2–35%) [26, 27]. Notably, the most frequent comorbidities were reported in SARS-CoV-2 patients treated with angiotensin-converting enzyme (ACE) inhibitors [27, 28]. SARS-CoV-2 binds to host cells through the ACE2 receptor, which is expressed by epithelial cells of the lungs, intestines, kidneys, brain, and blood vessels [29]. The expression of ACE2 is substantially increased in diabetic and hypertensive patients, treated with ACE inhibitors and angiotensin II type-I receptor blockers (ARBs) [29], which consequently promotes SARS-CoV-2 infection severity.

### Therapeutic Strategies for patients infected with SARS-CoV-2

No specific antiviral therapeutic agents or vaccine for SARS-CoV-2 are currently available to save the infected patients, protect health care workers and others at high risk of infection. Therefore, to control the rapidly growing SARS-CoV-2 outbreak, the WHO, announced on March 18, 2020, the launch of SOLIDARITY, which is an unprecedented multinational coordinated effort to collect rapidly robust clinical and scientific data during the SARS-CoV-2 pandemic [30], giving hope and planning to eradicate the SARS-CoV-2 virus. Various antiviral therapies with much broader landscapes are being selected by WHO, including the experimental antiviral drug Remdesivir; the Malaria medication Chloroquine/Hydroxychloroquine; a combination of Human Immunodeficiency Viruses (HIV) drugs such as Lopinavir and Ritonavir; and finally, a combination of HIV drugs added to Interferon-beta.

Remdesivir was originally developed by Gilead Sciences to combat Ebola and other related viruses by inhibiting viral replication. Remdesivir is an adenosine analogue with broad-spectrum antiviral activities [31]. A nucleoside analogue competes with natural nucleosides during replication for the RdRp active site, thus inhibiting the viral replication [32]. This drug is currently being extensively evaluated against SARS-CoV-2 in the United States and Europe, and according to the latest information, the efficacy of Remdesivir is found ambiguous against severely infected patients [33]. Despite its controversial results, the US Food and Drug Administration (FDA) approved the emergency use of the experimental Remdesivir to treat hospitalized SARS-CoV-2 patients [34].

Chloroquine and hydroxy-chloroquine have received intense attention worldwide because of the positive results generated from the preliminary studies of their use to treat SARS-CoV-2 patients. Chloroquine and hydroxychloroquine possibly decrease acidity in endosomes compartments of infected cells and can inactivate the virus (Fig. 3). In addition, chloroquine and hydroxy-chloroquine can also impair the terminal glycosylation of the ACE2 receptor, thus inhibiting the viral penetration into the cells [35]. However, ex-vivo studies performed in the cell culture model have suggested that chloroquine and hydroxy-chloroquine can cripple the SARS-CoV-2 virus, but the effective dose required is usually high, which can cause severe toxicity [36]. After reviewing the safety concerns of antimalarial drugs [37], the WHO temporarily suspended the hydroxy-chloroquine arm of its Solidarity trial [38].

Another underway ‘SOLIDARITY’ trial for SARS-CoV-2 treatment combines two drugs, Lopinavir and Ritonavir. These drugs were originally developed to treat HIV patients by inhibiting the protease enzyme that is needed by the virus to cleave long polypeptides chains during the assembly of new viruses [39]. Lopinavir and Ritonavir effectively inhibit the 3C-like proteinase, which plays a key role in the processing of viral polyproteins [40] and posing a possible potent therapeutic option against SARS-CoV-2. Although the preliminary data from the Chinese study is unclear [41], other clinical trials are underway (Table 1). In addition to the known antiviral drug combinations, some trials are currently exploring these drugs in combination with the anti-interferon-beta, an anti-inflammatory molecule [42].

**Table 1:** Emerging investigational therapeutic trials to treat SARS-CoV-2 patients

Drugs	Company	MoA	Comment/status
Remdesivir	Gilead Sciences, Inc.	Viral transcription inhibitor	Originally developed for Ebola and MERS Remdesivir found ambiguous against SARS-CoV-2
Chloroquine/hydroxychloroquine and Azithromycin combination	Novartis pharmaceuticals, Mylan NV, Teva Pharma-ceuticals USA	Decrease acidity in endosomes	Developed for malaria and rheumatoid arthritis SARS-CoV-2 patients showed substantial improvements after treatment WHO suspended the trial of hydroxychloroquine over safety concerns
Lopinavir-ritonavir, Kaletra	Abbott Laboratories, AbbVie, Inc.	Protease inhibitor	Anti-HIV treatment Initial clinical data indicates, no change in time to clinical improvement of SARS-CoV-2 patients
Lopinavir-ritonavir plus IFN-β	The University of Hong Kong	Protease inhibitor plus anti-inflammatory	IFN-β used in regulating inflammation in lessened disease No clinical efficacy data yet, but some doctors feels, it might be risky for patients with severe SARS-CoV-2 patients
Favipiravir/Avigan	Fujifilm Toyama Clinical	Viral transcription inhibitor	Next generation flu drug Drug accelerate the viral clearance and improve the lung conditions
Hydroxychloroquine and Nitazoxanide Combination	Tanta University	Blocks maturation of the viral hemagglutinin	Nitazoxanide has broad-spectrum activity against helminthic, protozoal, and viruses Clinical trial not yet started
Nafamostat and camostat	Susanne Arnold, University of Kentucky, University Hospital Padova	Antagonist TMPRSS2, Block the entry of SARS-CoV-2	Nafamostat and camostat are approved in Japan for use against pancreatitis Drugs are currently in phase 2/3 clinical trial in different countries
Hydroxychloroquine and Famotidine	Northwell Health, NY, USA	Inhibit the entry of SARS-CoV-2	Drugs are currently in phase 3 clinical trial in USA
Ruxolitinib	Novartis Pharmaceuticals	Janus kinase (JAK) inhibitor	Ruxolitinib was developed for high-risk myelofibrosis No efficacy data available against SARS-CoV-2
Sarilumab and Tocilizumab	Assistance Publique—Hôpitaux de Paris	Antibodies to inhibit IL-6	Sarilumab originally developed for rheumatoid arthritis Clinical trial for SARS-CoV-2 not yet started
Itolizumab	Biocon Limited	Anti-CD6 IgG monoclonal antibody	Itolizumab originally developed for chronic plaque psoriasis Clinical trial for SARS-CoV-2 completed and treatment showed substantial improvement in moderate to severe SARS-CoV-2 patients
APN-01	Apeiron biologics	ACE inhibitor	Originally developed for SARS treatment Clinical trial for SARS-CoV-2 not yet started
siRNAs	Alnylam pharmaceuticals and vir biotechnology	Conserved regions of coronavirus RNA.	siRNAs hit highly conserved regions of SARS-CoV-2 RNA Clinical trial not yet started
N-803	ImmunityBio	Kill the infected cells	N-803 showed strong positive response in monkeys against HIV Clinical trial not yet started
Pirfenidone	Roche	Anti-inflammatory drug, inhibits transforming growth factor-β	Pirfenidone used for idiopathic pulmonary fibrosis (IPF), a lung fibrosis disease Pirfenidone can improve lung functions in SARS-CoV-2 patients No clear clinical efficacy data yet
Umifenovir	Shahid Beheshti University of Medical Sciences, Iran	Binds to viral lipid membrane and inhibits viral entry	Approved for influenza A and B virus in Russia and china Umifenovir found ambiguous against mild to moderate SARS-CoV-2
Ivermectin	Sidney Kimmel Comprehensive Cancer Center at Johns Hopkins	Destabilize the cell-transport proteins	Ivermectin is broad-spectrum anti-parasitic drug Administration of Ivermectin in SARS-CoC-2 patients found efficacious Drug is being tested in combination with hydroxy-chloroquine in many countries
Corticosteroids	Hospices Civils de Lyon, France	Anti-inflammatory	Corticosteroids are being tested in SARS-CoV-2 patients

Apart from the ‘SOLIDARITY’ trial, other therapeutic options are also being explored against SARS-CoV-2 to improve the outcomes of critically ill patients. As of Aug 12th, 2020, more than 1000 clinical trials are currently exploring different treatment strategies against SARS-CoV-2 [42], including drug repositioning, novel therapeutic options, and vaccines. Potential treatment strategies that are currently in the testing phase against SARS-CoV-2 or likely to be initiated as clinical trials are summarized in table-1. These include drugs that can reduce inflammation (such as itolizumab that binds to CD6 receptor and blocks the activation of T lymphocytes and suppress the pro-inflammatory cytokines or corticosteroids that decrease the cytokines storm), ACE-2 inhibitor, SARS-CoV-2 specific siRNAs, and immunomodulators. In addition, a number of reposition antiviral drugs such as Favipiravir (a nucleoside analogue inhibiting the RNA polymerase), ribavirin (a guanosine analogue), are also being tested against moderate to severe SARS-CoV-2 patients [43]. The potential viral targets and clinical status of these therapeutic options are shown in Fig. 3 and Table 1.

In addition to the antiviral treatment options, systemic transfusion of convalescent plasma collected from healthy donors who recovered from SARS CoV-2 is being tested in different clinical trials on severely infected SARS-Cov-2 patients to reduce the cytokines storm and to replenish the patient’s own antibodies during the acute phase of the disease. Interestingly, the administration of convalescent plasma containing neutralizing antibodies showed a significant decline in the viral load within few days post-transfusion and a substantial improvement in the clinical conditions of the patients [44]. Several companies and universities, such as Takeda, Mount Sinai, and Hopkins are evaluating the mass-production of monoclonal antibodies. Importantly, the success of the convalescent sera transfusion has given clues on how the immune system combats SARS-CoV-2, and how easily a vaccine can be made. In addition, virus-specific neutralizing antibodies that can accelerate the virus clearance and/or prevent its entry into target cells can serve as the primary mechanism for the restriction and clearance of the virus (Fig. 3).

### Vaccine for SARS-CoV-2

With the challenges known to be associated with generating a vaccine against RNA viruses, experts feel that developing an efficacious vaccine for SARS-CoV-2 will be very challenging [45]. RNA viruses are known to be difficult when it comes to vaccine development; however, more than 100 research groups, including biotech companies and research institutes, are currently evaluating different approaches [46]. While some of these vaccines have initiated human trials [44] (Table 2), according to the latest data, some vaccine candidates such as ChAdOxa nCoV-19 (containing spike protein to boost antibodies production against spike protein), and Gam-COVID-Vac Lyo have shown an effective single-dose immune response in clinical trials [44]. However, most experts estimate that a successful vaccine will not be available before 2021.

**Table 2:** Emerging investigational vaccine trials to treat SARS-CoV-2 patients

Vaccines	Company	MoA	Comment/status
Targeted SARS-CoV-19 antibodies	Tsinghua University in Beijing, China	Neutralize SARS-CoV-2 virion to infect cells	Specific antibodies can inactivate the viral particle, which eventually could be helpful in treating COVID-19 patients No clinical trial yet
ChAdOxa nCoV-19 vaccine	Oxford University	Immune system	Spike protein of SARS-CoV-2 expressed in harmless common cold adenovirus Vaccine candidate showed effective immune response in clinical trial
Gam-COVID-Vac Lyo	Gamaleya Research Institute, Russia	Immune system	Gam-COVID-Vac Lyo”, is a viral vector-based vaccine-fused with the spike protein of SARS-CoV-2 to stimulate the immune response Vaccine showed effective single-dose immune response in clinical trial
mRNA-1273	Moderna, Inc.	Binds to SARS-CoV-2 RNA	Entered in clinical testing Phase-2 No clinical efficacy data yet
DNA vaccines	Inovio	Immune system	DNA plasmid expressing S (spike) protein Presently at phase-1 trial
Virus-like particles	CanSino Biologicals	Immune system	Developing vaccine by expressing S (spike) protein in adenovirus Presently at the pre-clinical stage
Live attenuated vaccine	Soligenix and University of Hawaii	Immune system	Live inactivated vaccines are challenging to grow and scale-up Presently at pre-clinical stage
Repurposed vaccines Bacille Calmette-Guerin	Assiut University	Immune system	Trial underway against SARS-CoV-2 No clinical data yet

The development of an effective vaccine is the ultimate solution to control this battle; however, once a safe and potent vaccine becomes available, it will be vital to make it accessible to everyone who needs it. Therefore, regulatory agencies need to align with R & D to fast-track the pre-clinical and clinical evaluation, regulatory approvals and mass production of vaccine for worldwide distribution for all populations.

Although several repositioned drugs are being tested against SARS-CoV-2 and most of these drugs have already been approved for another disease. This indicates that these drugs do not act specifically against human SARS-CoV-2 and have not been tested against COVID-19 in animal models, though that would usually require FDA approvals. Another factor should also be considered: the clinical trials performed to get approval for other diseases often does not evaluate combinations with other drugs. So, we feel that the potential for synergistic toxicity needs to be evaluated before such ‘repositioned’ drugs approved for SARS-CoV-2 treatment regimes. Considering the pandemic situation, evaluation of therapeutic molecules and vaccine candidates against this emerging infection is a crucial step in the management of SARS-CoV-2 disease, which seems to be key in combating pandemics; however, regulatory agencies must require to check with developers of vaccine candidates and repositioned drugs for potential efficacy and safety evaluation in animal studies.

## Conclusions

Despite the fact that the number of new SARS-CoV-2 cases have started to slow down in many countries, health experts and epidemiologists are warning that we are still in the early stages of the pandemic. A complete return to normal life will only be possible once a vaccine is found and made available to everyone, which seems still at a very early stage of development and will require more time. In the meantime, some effective therapeutic options are urgently required to control the COVID-19 pandemic and give hope to save human lives. We have highlighted here the current status of the therapeutic tools used in the battle against COVID-19. While FDA is approving different clinical trials to fast-track the efficacy assessments of different anti-viral drugs or drugs combination, gathering quality clinical data will be vital to ensure the safety and efficacy. The current battle against COVID-19 pandemic also emphasizes the need for policies for being better equipped for any future pandemic, which includes increased funding to drugs and vaccines development, kits development, testing facilities, and fast-track FDA approval policies.

## Supplementary information

**Additional file 1: Figure S1.** Worldwide reported SARS-CoV-19 cases and deaths. Top 10 massively impacted countries by SARA-Cov-19 (A). Worldwide infection and mortality graph of SARS-CoV-2 (B), estimated infection rates of common viral outbreaks (C).

## Data Availability

Not applicable

## Abbreviations

COVID-19: Coronavirus disease of 2019
CoV: Coronavirus
SARS-CoV-2: Severe acute respiratory syndrome coronavirus 2
WHO: World Health Organization
CI: Confidence interval
MERS-CoV: Middle East Respiratory Syndrome Coronavirus
SARS-CoV: Severe acute respiratory syndrome coronavirus
CFR: Case fatality rate
ORFs: Open reading frames
S: Spike
E: Envelope
M: Membrane
N: Nucleocapsid
ACE: Angiotensin-converting enzyme
ACE2: Angiotensin-converting anzyme 2
TMPRSS2: Transmembrane protease serine-2
RCT: Replication transcription complex
ARBs: Angiotensin II type-I receptor blockers
HIV: Human Immunodeficiency Viruses
FDA: Food and Drug Administration
