# Supplementary material for: Pathophysiology and treatment strategies for COVID-19
Source: J Transl Med. 2020 Sep 15;18:353. doi: 10.1186/s12967-020-02520-8 (PMC7491044; doi:10.1186/s12967-020-02520-8)
Supplement: Supplementary file 1 — Additional file 1: Figure S1. Worldwide reported SARS-CoV-19 cases and deaths. Top 10 massively impacted countries by SARA-Cov-19 (A). Worldwide infection and mortality graph of SARS-CoV-2 (B), estimated infection rates of common viral outbreaks (C). [file 12967_2020_2520_MOESM1_ESM.docx]

**Figure S1.** Worldwide reported SARS-CoV-19 cases and deaths. Top 10 massively impacted countries by SARA-Cov-19 (A). Worldwide infection and mortality graph of SARS-CoV-2 (B), estimated infection rates of common viral outbreaks (C).
